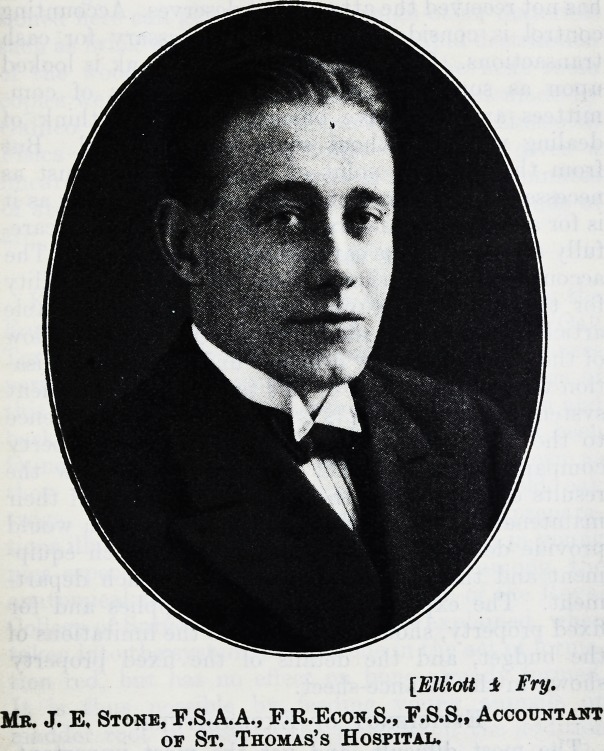# Accounting in Relation to Hospital Administration
*A paper read at the first annual Conference of the Incorporated Association of Hospital Officers.


**Published:** 1924-06

**Authors:** Joseph E. Stone


### ACCOUNTING IN RELATION TO HOSPITAL ADMINISTRATION.*

By JOSEPH E. STONE, F.S.A.A., F.S.S., F.R.Econ.S.

Until comparatively recent times the faithful recording of cash transactions and the preparation of summaries of these transactions for the annual statements of account were thought to be the highest point of development in hospital accounting. The increasing size and complexity of modern hospital business transactions has, however, caused a new orientation in hospital administration. The analytical method will not supplant the synthetic method employed by the old-time officer. The need for a strong leader to direct the policy of the hospital is greater than ever, but he will now have recourse to reliable data provided by the various analytical functions of which accounting may be said to be one of the most important.

[Elliott & Fry.

Mr. J. E. Stone, F.S.A.A., F.R.Econ.S., F.S.S., Accountant of St. Thomas's Hospital.

### Definition and Objects of Hospital Accounting.

Hospital accounting may be defined as a method employed by a hospital for the purpose of recording its external and internal business transactions, and finally co-ordinating these data around problems of administration. Its object is to express clearly by comparative analysis the actual effect of the transactions, and to aim at the presentation of interim results in such a manner that they will enable the administration to manage efficiently and economically the various activities maintained, and to provide data that will act as a guide in connection with future transactions of a similar nature. A discussion of the subject of this paper involves a consideration of the system of accounting now in use. This system is known as "The Revised Uniform System of Accounts for Hospitals." Under it, expenditure is divided into a number of main headings, and these are again divided into sub-headings showing details. Each main head represents a specific group of expenditure, and within the limits of each of these and the subheads the particulars of expenditure must be set out with minute accuracy. Analysis of expenditure must conform exactly to these headings, and this strict adherence to form is a sacred rule of hospital accounting.

### Deficiencies of the Old System.

Summarising briefly the deficiencies of the present system from the administrative point of view, they are as follow :—It does not show the cost of operating any one of the many and varied activities maintained. It does not differentiate between the cost of services included in the same head or sub-head of expenditure. It does not furnish any material for the comparison of cost of administering similar services. It does not assist in the elucidation of the problems of administration, because it does not show who is responsible for expenditure. It does not lend itself to the establishing of units of costs in respect of the activities. It does not take into account the residual value of the expenditure. It provides at best merely a summary of totals unrelated to any activity. It does not show the cost of the staff as distinct from patients. Expenditure cannot be reduced scientifically because the accounts do not show which costs are high in proportion to the services rendered.

### The New System.

The difference between the new system and the one at present in use is the difference between the subjective and the objective methods of classifying expenditure, and it can best be emphasised by an illustration. Linen purchased in bulk and stored for subsequent use may be used for many different services. Some may go to medical wards, to surgical wards, to special departments, and some to staff quarters. The assistant whose duty it is to analyse the accounts cannot tell for what services the linen will be used, but he can see from the account the nature of the goods purchased, and he has no difficulty in classif ing it as "domestic," renewals and repairs of bedding and linen, and he records the amount under this heading. This is known as the subjective method, and it is the basis of the present system.

### Principles of the Accounting System.

Given an organisation that can be held accountable, the next consideration is the introduction of a system of accounts which will hold the organisation accountable. The value of the accounting system lies in its value in increasing the efficiency of the administration. Special emphasis is placed on this, as, under

* A paper read at the first annual Conference of the Incorporated Association of Hospital Officers.

the present system, the accounts have no relation to the organisation, and are generally regarded as an end in themselves. Hospital accounts, then, if they are to serve the purpose for which they are intended, must conform to the following principles:—1. An organisation chart showing all the units of the organisation should be the starting point of the system. 2. The introduction of a hospital budget based on this chart. 3. The inception of a separate account for each unit of the organisation. Within each account the analysis of expenditure to be grouped under appropriate headings distinguishing between that which is controllable and that which is uncontrollable. 4. The reduction of the total expenditure in each account to a unit of cost, each unit being determined solely by the nature of the service rendered. 5. The system must be based on pure expenditure lines representing the true cost for the period under review.

### The Unit Accounts.

It follows naturally that an accounting system is a prerequisite to the preparation and enforcement of a hospital budget. After the budget is prepared it must be enforced, and a system of accounts is necessary for such enforcement. It is idle to allot a certain amount of funds to a department if no record is kept of its expenditure so that they will be restricted to the allotment. The opening of a separate account for each unit allows the system to harmonise with the form of the budget, and we see here the inseparable relation between organisation, accounting and budget. The responsible officers may be informed continuously in terms of £. s. d. the cost to the hospital of what they are doing. Apart from the silent control invariably introduced by the figures themselves, they also help to develop the money sense in administrative officers.

### The Income and Expenditure System.

The fourth principle involves another fundamental change in the present system. With the increase in knowledge of accounting it is unnecessary to explain at length the inadequacy of the cash-receipts-and-payment system as a basis for exercising administrative control. Knowledge of the true position is required, and this can only be provided by the income and expenditure system, which includes such factors as consumption of stores, outstanding expenditure, assets and liabilities, etc. The maintenance of accounts of true expenditure (as distinct from cash payments) is the method adopted by all commercial undertakings, not because they are commercial undertakings, but because it is recognised that it is the correct and most efficient method for purposes of control. The putting into practice of the principles enunciated above would result in a more highly developed form of accounting for hospitals, and one less influenced by mere convention. Each unit of the organisation would show its own total cost and its unit of cost.

### Up-to-Date Principles.

Other considerations make it equally desirable that hospital accounts should be based upon more up-to-date principles. The Ministry of Health requires the submission of detailed statements of expenditure on venereal diseases, and on maternity and child welfare departments in connection with claims for grants. With the growth in hospital activities this practice will grow. Again, one looks in vain in the published reports of hospitals for any account of the cost incurred in maintaining such valuable subsidiary departments as X-rays, electrical treatment, massage, research, etc. These reports appear year after year in the same form with only the figures altered; whereas, by inserting figures relating to the activities conducted, emphasising those to which it is desired to direct special attention, and informing the public of the extent of the research work carried on, and the cost, they could be transformed into very effective collectors.

### Hospital Property.

This is a phase of hospital administration which has not received the attention it deserves. Accounting control is considered absolutely necessary for cash transactions. Cash in hand or at the bank is looked upon as something sacred, and members of committees and executive officers would not think of dealing with it without some form of ritual. But from the financial point of view control is just as necessary for the correct utilisation of the stores as it is for actual cash, and exactly the same rules, so carefully observed in the care of cash, should apply. The accounting records should place definite responsibility for the use of all supplies and readily consumable articles, and should show comparative data to allow of the consumption by different units of the organisation to be obtained. For this to be done an efficient system of stores accounts is essential. With reference to the more permanent forms of hospital property comparative data should be available to show the results obtained from their use and the cost of their maintenance. A record should be kept which would provide detailed data with reference to such equipment and the equipment employed in each department. The expenditure, both for supplies and for fixed property, should be subject to the limitations of the budget, and the details of the fixed property shown in the balance-sheet.

### Efficiency of Expenditure.

The most difficult, and yet the most important, task of a hospital administrator is the showing of efficiency in expenditure. If the accounting system provides for a departmental system of classification of expenditure and a classification within each department by subjects, it is possible to obtain expenditure by activities. Unit costs may then be calculated for each. The limitations of such costs as a final index of efficiency are not lost sight of, but they serve a valuable purpose in showing trends and as tentative standards by which the efficiency of activities may be judged. An increase in expenditure is not in itself an indication of poor efficiency or lack of economy, but when expenditures are reduced to units reliable comparisons may be made, and fluctuations inquired into, not annually, but continuously, month by month. The final test of the value of a system of accounts for hospitals lies in its success in multiplying the power and increasing the effectiveness of the administration. Viewed thus, it will be seen that accounting is a vital factor in hospital administration, and it should be developed along the lines of administrative procedure.

## Figures and Tables

**Figure f1:**